# Multi-factor settlement prediction around foundation pit based on SSA-gradient descent model

**DOI:** 10.1038/s41598-022-24232-3

**Published:** 2022-11-17

**Authors:** Zhengcai Li, Xinmin Hu, Chun Chen, Chenyang Liu, Yalu Han, Yuanfeng Yu, Lizhi Du

**Affiliations:** 1 College of Construction Engineering, Jilin University, Changchun, 130026 China; 2 Beijing Aidi Geological Engineering Technology Co., Ltd, Beijing, 100000 China

**Keywords:** Civil engineering, Scientific data

## Abstract

With the rise of machine learning, a lot of excellent algorithms are used for settlement prediction. Backpropagation (BP) and Elman are two typical algorithms based on gradient descent, but their performance is greatly affected by the random selection of initial weights and thresholds, so this paper chooses Sparrow Search Algorithm (SSA) to build joint model. Then, two sets of land subsidence monitoring data generated during the excavation of a foundation pit in South China are used for analysis and verification. The results show that the optimization effect of SSA on the gradient descent model is remarkable and the stability of the model is improved to a certain extent. After that, SSA is compared with GA and PSO algorithms, and the comparison shows that SSA has higher optimization efficiency. Finally, select SSA-KELM, SSA-LSSVM and SSA-BP for further comparison and it proves that the optimization efficiency of SSA for BP is higher than other kind of neural network. At the same time, it also shows that the seven influencing factors selected in this paper are feasible as the input variables of the model, which is consistent with the conclusion drawn by the grey relational analysis.

## Introduction

Considering that the utilization of surface space is close to saturation, an increasing number of functional buildings are developing in the direction of super large and high, so deep foundation pit projects are inevitable^1–3^. During construction, land subsidence has a huge impact on the construction safety and surrounding buildings, simultaneously, because of various sources of uncertainties, it’s hard to predict accurately^4,5^. However, many studies show that the settlement around the foundation pit is affected by multiple factors which including physical parameters of soil and external conditions during construction^6,7^. Then, it is of great significance to establish a model that can map the potential nonlinear relationship and provide reference for subsequent safe construction^8,9^. In the past ten years, the prediction of land subsidence has attracted the attention of many scholars in geotechnical engineering. In 2014, Su.et al. used Kalman filter in a subsidence monitoring method and analyzed it by means of forward modeling, the result shows that it is feasible to predict the settlement of the subsequent construction by training the data the previous stage^10^. In 2017, Nejad and Jaksa proposed a supervised learning algorithm that uses the CPT data for the load settlement simulation, however, excessive input variables will seriously affect the speed and accuracy of the network training and decrease the generalization ability^11^. In 2017, Cao et al. explored the influence of different input variables on settlement through the parameter sensitivity analysis formula, and the study proved that a single variable cannot well explain the settlement results^12^. And related research shows that when linear loading conditions are met, the subsidence and excavation time can be fitted to an S-shaped curve^13^, in view of the non-linear characteristics of foundation pit settlement, so it’s crucial to select suitable nonlinear mapping models^14^. Some researchers choose BP, Support Vector Machine (SVM) and gray Verhulst models^14–16^ to predict the foundation settlement. Compared with traditional methods, these nonlinear prediction models have better performance. However, due to the lack of self-learning and error correction capabilities, when the short-term settlement data fluctuates greatly, the gray Verhulst model is not stable in the prediction^17^. For the limitation of insufficient sample size and weak linear feature performance, the SVM model can exert its unique advantages, but it also has some shortcomings that are sensitive to the choice of parameters and kernel functions^18^. Since BP was proposed in 1986, it has been successfully used in various engineering fields with its powerful self-learning, nonlinear mapping and error feedback adjustment capabilities^19^. However, like most neural networks that use gradient descent to optimize parameters in a negative feedback process, the random selection of initial weights and thresholds greatly affects the prediction performance of BP. This also makes it difficult for the BP algorithm to achieve the overall optimum, but tends to converge to a local minimum point, and the convergence speed is also slow^20,21^. In 1991, J. L. Elman established the Elman model to solve some speech processing problems^22^. Guo et al. applied Elman to the deformation prediction of foundation pit, and it showed that the prediction accuracy was high, but the sample size was too small and it is difficult to jump out of the local optimal solution^23^.

SSA is a swarm intelligence optimization algorithm that was proposed in 2020, which can optimize the mapping relationship between the input and output variables of the prediction model^24^. SSA can efficiently optimize the weights and thresholds of BP and Elman, and improve the prediction accuracy of the model. That's why it was chosen.

## Prior knowledge needed to know

**Figure 1:**
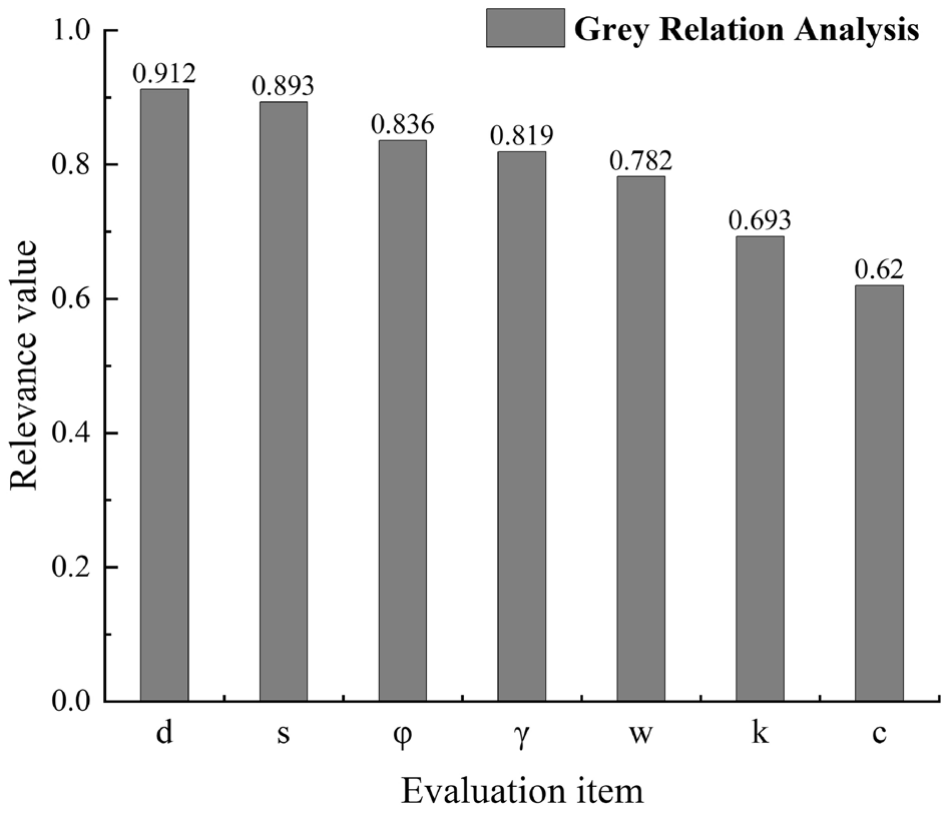
Grey relation analysis value.

In the previous research, only a single influencing factor of time was usually considered, and the unit was weeks or months. However, setting a larger time unit will lose the engineering significance of prediction, and has little guiding significance. Therefore, this paper selects two groups of continuous monitoring data whose time unit is day, and each group has 170 pieces of data. These data are arranged strictly according to the time of excavation, so they cannot be disrupted during machine learning training, this is different from the random shuffling of the data set in general machine learning. Feng et al. show that factors including excavation depth, the number of internal supports, etc. can affect the settlement of the foundation pit^25^. And during excavation, when the soil geological conditions are good, the land subsidence is relatively small, while the subsidence may be relatively large when it’s poor^26^. The reason that the previous studies seldom consider the soil’s mechanical parameters is that it’s impractical to record the daily excavation depth and the daily soil type. But, in this paper, first, timely and accurately record the specific time and corresponding level information of each support when it is arranged. Then, the daily excavation depth can be measured by the support elevation, or the two adjacent support elevations and their excavation time spans can be recorded first and then interpolated. Finally, according to the geological data obtained from on-site drilling and the above-mentioned data of excavation time and depth recorded accurately every day, the soil type and parameters for real-time excavation can be determined. Obviously, the supports also have a certain influence on the settlement, so the number of supports is used as an input variable in this paper. In addition, there also attempts to use the groundwater level, soil permeability, internal friction angle, gravity and cohesion as input variables. In order to make the settlement prediction of the model more accurate, the input variables of the model should take into account the potential factors affecting settlement as much as possible. Therefore, this paper conducts Grey relation analysis on the original data set. The basic idea of grey relational analysis is to determine whether the relation between each column of data to be evaluated and the parent sequence is close by determining the relational value. The analysis results are shown in Fig. 1.

After the grey relation analysis, factors with the correlation value greater than 0.6, indicating higher correlation degree, were selected as the input variables. In Fig. 1, *d*, *s*, *φ*, *γ*, *w*, *k* and *c* represent the excavation depth, the number of internal supports, the internal friction angle, soil gravity, the groundwater level, the permeability coefficient and cohesion, respectively.

## Method

### Introduction to BP and Elman neural network

**Figure 2:**
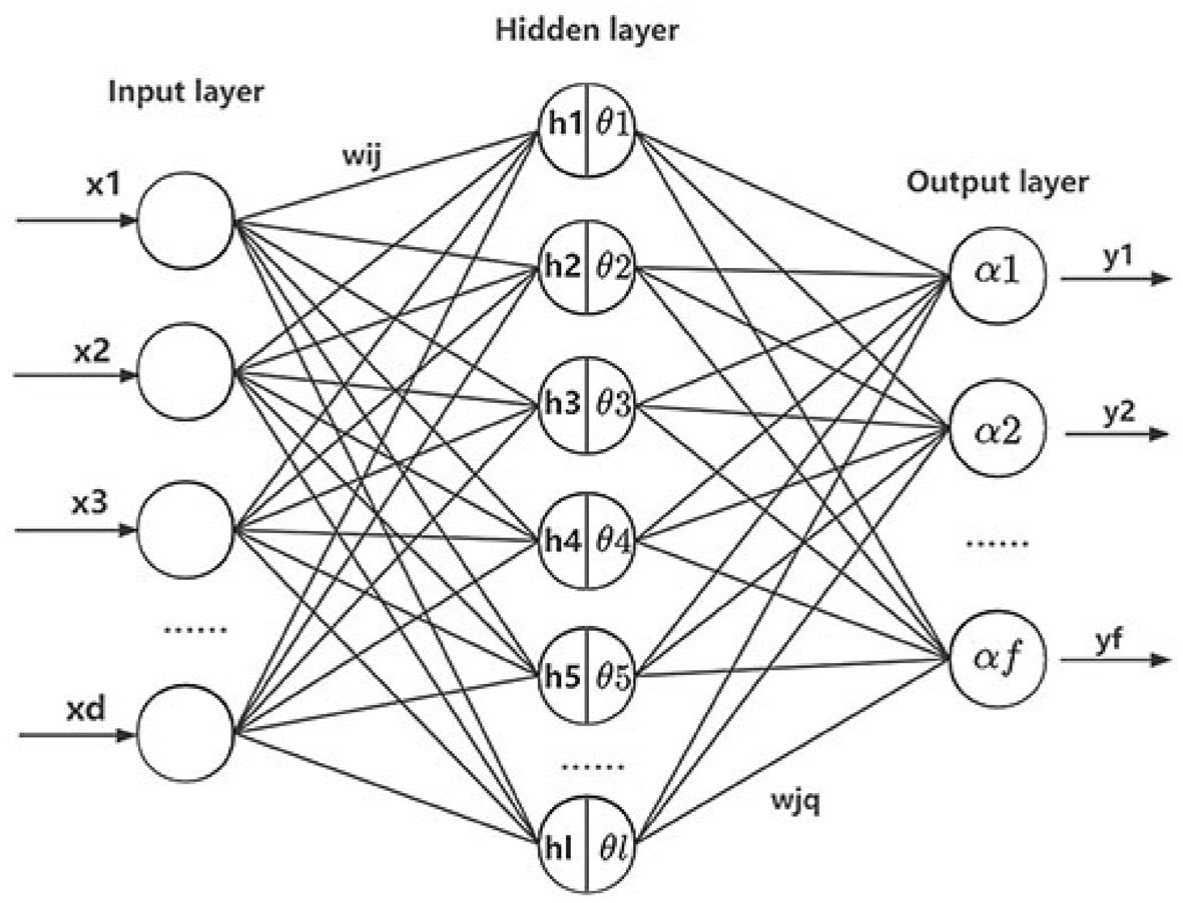
Basic structure diagram of BP neural network.

BP is multi-layer feed-forward network, error back propagation is the meaning of the name of BP. In effect, it is a linear or non-linear mapping of the relationship between input variables and output variables. Mathematical derivation proves that a three-layer neural network structure can approximate any continuous function within acceptable accuracy. The first stage of the BP is that the training samples are propagated through the input layer and the hidden layer, then output layer gets the corresponding output, the second stage is the back propagation of the error, and the third stage is the weights and thresholds update until the end condition is met^27–29^. Figure 2 shows its basic structure. Equation (1) is its mathematical calculation expression:

1 $$ E = \frac{1}{2}\sum\limits_{q = 1}^{f} {\left( {Y_{Q} - C_{Q} } \right)}^{2} = \frac{1}{2}\sum\limits_{q = 1}^{f} {\left( {N\left( {\sum\limits_{j = 1}^{l} {\left( {w_{jq} F\left( {\sum\limits_{i = 1}^{d} {\left( {w_{ij} - \theta_{j} } \right)} } \right) - \alpha_{q} } \right)} } \right) - C_{Q} } \right)}^{2} $$

According to Eq. (1), the error *E* can be reduced by updating the weights and thresholds, and this is where SSA comes into play.

**Figure 3:**
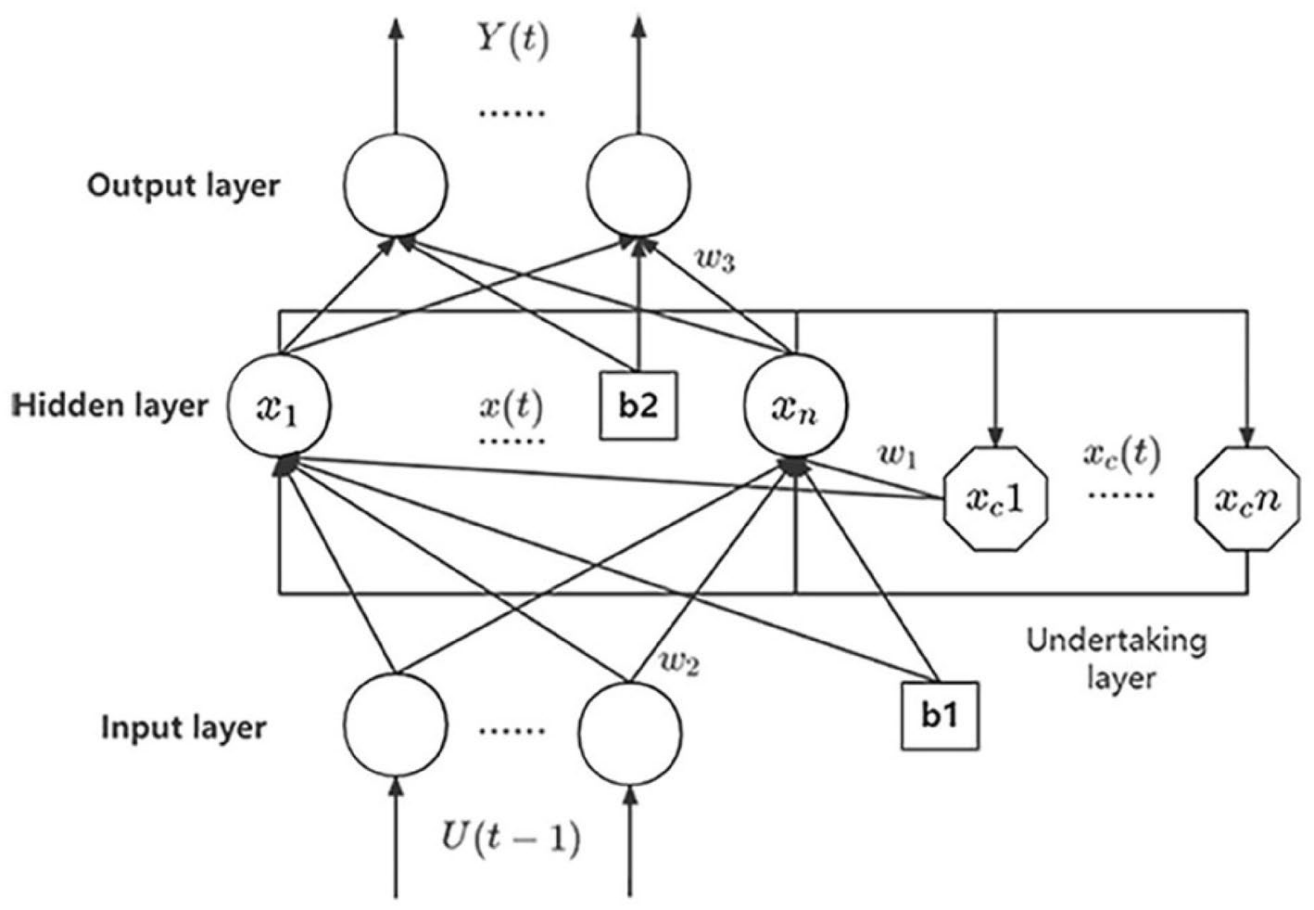
Simple structure diagram of Elman.

Elman is a typical dynamic neural network, compared with BP, it adds an undertaking layer with feedback function to the hidden layer, and it also has better prediction accuracy, so it’s more suitable for the prediction of foundation pit^30^. However, like BP, Elman is also based on gradient descent to reduce the error, so the training of the model tends to fall into local optimum but not global optimum^31^. Figure 3 is its basic structure.

The Elman learning indicator function also uses the error sum of squares function:

2 $$ E(\omega ) = \sum\limits_{t = 1}^{n} {\left( {Y_{t} \left( \omega \right) - Y_{t}^{^{\prime}} \left( \omega \right)} \right)}^{2} $$

where $Y_{t} \left( \omega \right)$ and $Y_{t}^{^{\prime}} \left( \omega \right)$ represent the output value and the expected value, respectively.

### Introduction to Sparrow Search Algorithm

Inspired by the foraging and anti-predation behaviours of sparrow populations in nature, in 2020, Xue and Shen proposed SSA, idealizing the following behaviours of sparrows and briefly formulate corresponding rules to understand the process of sparrow optimization clearly^32^.

- Rule (1). Discoverers usually has the ability to expand the scope of the food search and provide real-time regional location information to all other joiners, The higher the fitness value in the model, the higher the energy reserve of the sparrow.
- Ruler (2). When sparrows find danger, they will send out an alarm signal, and when the alarm value exceeds the safety threshold, the discoverers will take joiners to other areas.
- Rule (3). The ratio of discoverers to joiners in the entire population is constant, but as long as a richer source of food can be found, every sparrow can become a discover, but if one sparrow becomes a discoverer, another sparrow must become a joiner.
- Rule (4). Joiners with poor fitness have poorer foraging positions in the population, and naturally, they are more likely to fly away from these places.
- Rule (5). During foraging, discoverers with better food resources will always attract those discoverers to grab food from them or to forage around them.
- Rule (6). When the feeding area in which the sparrows are no longer safe, they will quickly move away from the danger area, and sparrows that do not feel danger will walk randomly to get close to other sparrows.

### Establishment of the joint models

Based on the above idealized model, the SSA optimization process is as follows: (1) Eliminate abnormal data in the data set, and then select variables that have a greater impact on settlement through grey correlation analysis. (2) Establish the initial network structure and select the appropriate transfer function. (3) Select SSA parameters, including fitness function, population size, proportion of sparrows and maximum number of iterations. (4–5) Calculate and find the best fitness value of the sparrow and its corresponding global best position, and then update the positions of the three kinds of sparrows in the population (6) Determine whether the end condition is met, if not, go back to (4–5), if satisfied, go to (7). (7) The optimal thresholds and weights obtained by SSA are assigned to the initial model.

**Figure 4:**
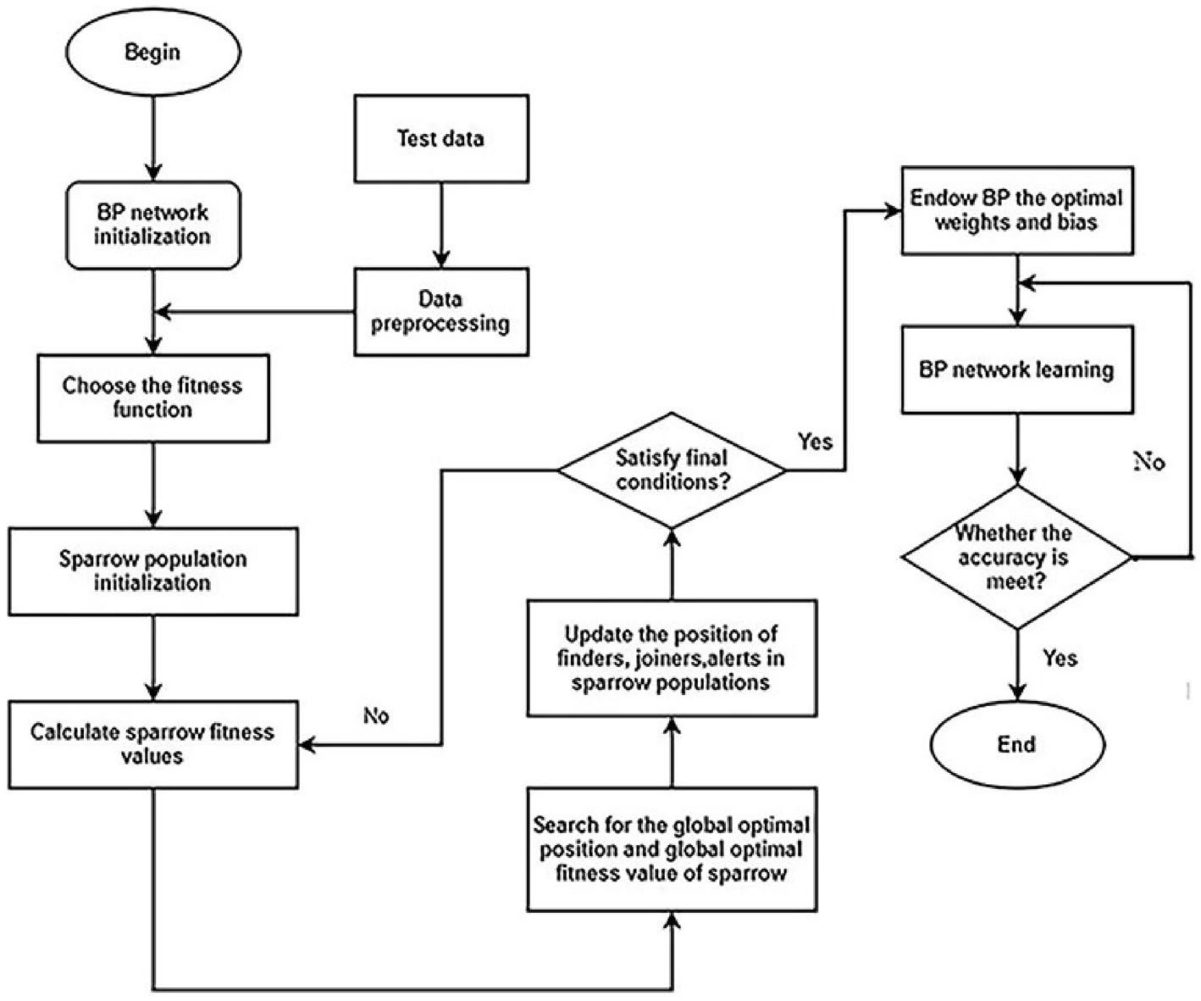
Flow chart of SSA optimizing BP.

**Figure 5:**
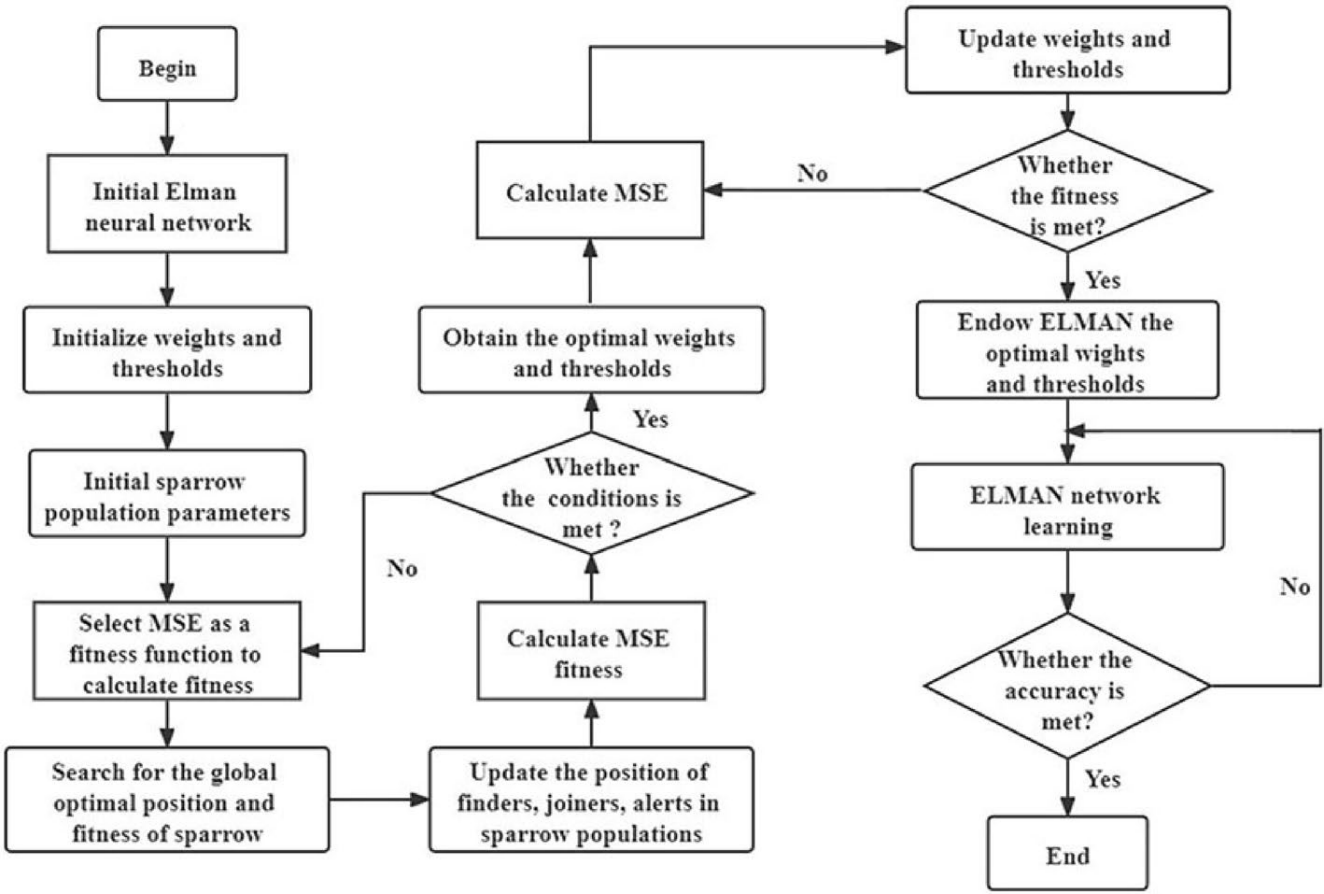
Flow chart of SSA optimizing Elman.

Figures 4 and 5 are the simple flow charts of SSA optimizing BP and Elman, respectively.

## Engineering examples and parameter selection

### Brief description of the project

**Figure 6:**
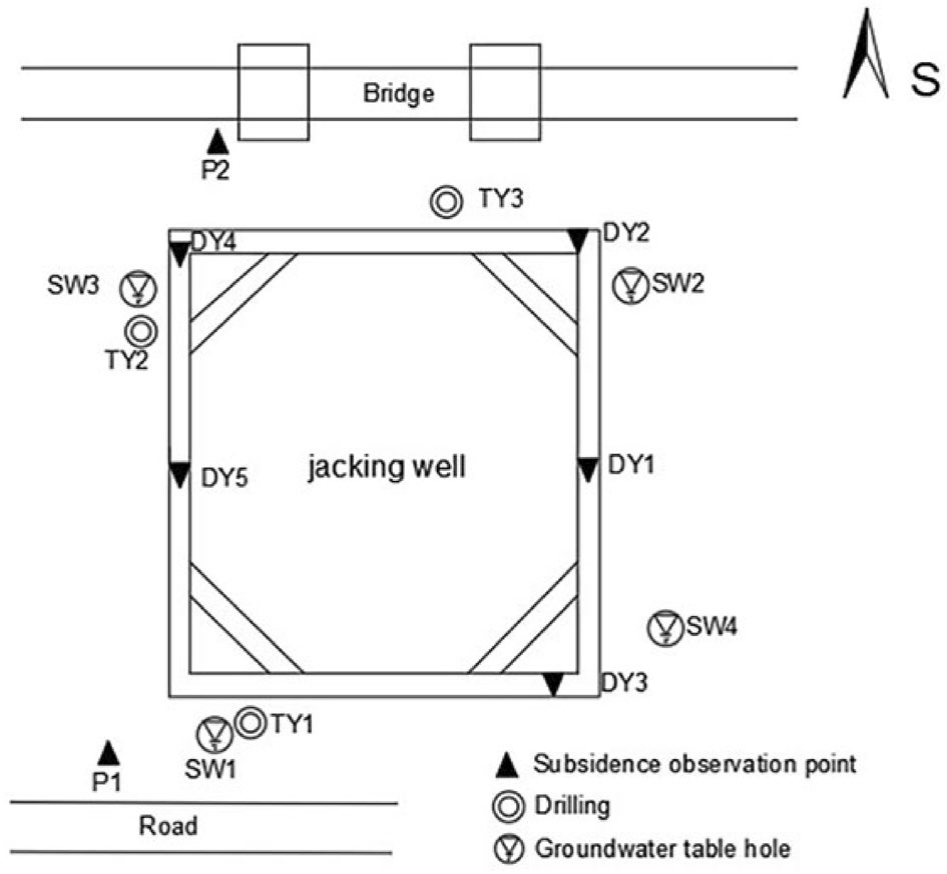
Layout of settlement monitoring points around foundation pit.

The foundation pit is located in a city in South China, and as shown in Fig. 6, the settlement monitoring point *P*1 is close to the road and *P*2 is close to the bridge. Excessive settlement will affect driving and building safety therefore, so it is of great importance to predict settlement. At the same time, the foundation pit is located in an area with heavy rainfall. Therefore, the influence of the groundwater level around the foundation pit cannot be ignored. *SW*1, *SW*2, *SW*3, *SW*4 represent four groundwater level monitoring points, in this paper, the water level values near the monitoring points *P*1 and *P*2 are taken respectively.

**Figure 7:**
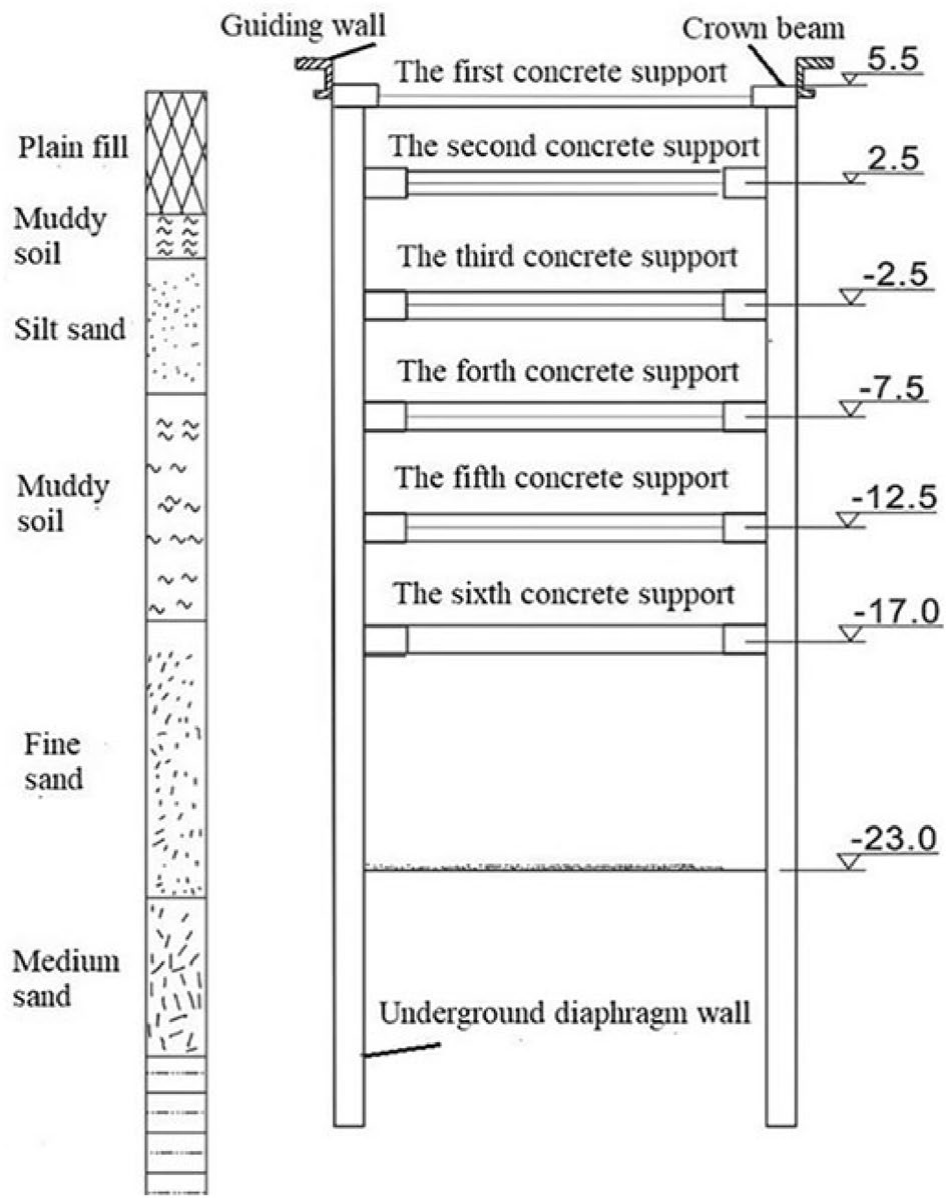
Schematic diagram of soil layer distribution and support structure.

The soil layer distribution and elevation of the supporting structure are shown in Fig. 7. There are five layers in total until the excavation depth, which are plain fill, muddy soil, silt sand, muddy soil and fine sand, respectively.

By analyzing the samples collected along different drilling depths, combined with geological exploration and drilling data, the thickness of each soil layer and the physical properties such as the internal friction angle, weight and cohesion of the soil can be obtained. Table 1 lists the relevant parameters of each soil layer. The survey data also show that the foundation pit and surrounding soil layers are relatively uniform, and there are no active faults in the affected area. Therefore, the inhomogeneity of the geological structure and the anisotropy of the foundation pit structure are not considered when selecting the input parameters.

**Table 1:** Soil layer parameter table.

Soil type	Internal friction angle (°)	Soil weight (KN. m^−3^)	Cohesion (kPa)	Permeability coefficient (cm. s^−1^)
Plain fill	12.0	19.0	6.00	5.96E2
Muddy soil	6.20	17.2	9.66	4.32
Silt sand	23.0	18.0	2.00	6.48E2
Fine sand	29.0	19.0	0.00	5.441E3
Medium sand	30.0	19.5	0.00	1.667E4

### Model dataset creation

The subsidence data of the two monitoring points *P*1 and *P*2 were selected, each group has 170 sets of data, the time unit is days, and both of datasets are 8-dimensional, the first 7 dimensions are input variables, the last dimension is output variables. The dataset cannot be shuffled in this paper, selecting data with this rule reduces the prediction accuracy of the model to a certain extent, because it cannot fully learn the characteristics of the test set, but it must be considered that these settlement data are sorted in time series. Therefore, the 150 pieces of data in the training set and the 20 pieces of data in the test set are chosen strictly in order.

### Model parameter settings

The models are trained in MATLAB in this paper. In BP model, the input layer, hidden layer, and output layer select "tansig", "logsig", and "purelin" function, respectively. The nodes number *n* of hidden layer can be roughly determined according to empirical Eq. (3).

3 $$ n = \sqrt {d + l} + \sigma $$

where *d* is the dimension of the input layer, *d* = 7; *l* is the number of nodes in the output layer, and *l* = 1, $\sigma$ is a natural number from 0 to 10.

The Elman model is a four-layer structure, compared with BP, it adds a layer of undertake layer, the number of nodes in the input and output layers is also the same as BP, the number of hidden layer nodes can also refer to Eq. (3).

The population size and evolution number of sparrows were chosen to be 20 and 30, respectively, and the number of discoverers was set to 20% of the total.

## Model performance comparison and analysis

### Performance analysis of the joint model under the P1 monitoring point

The solution of the evolutionary computing method will be different each time, so this paper will make multiple consecutive predictions of a model at the same monitoring point, and then calculate the mean predicted value, MSE and predicted value variance.

**Figure 8:**
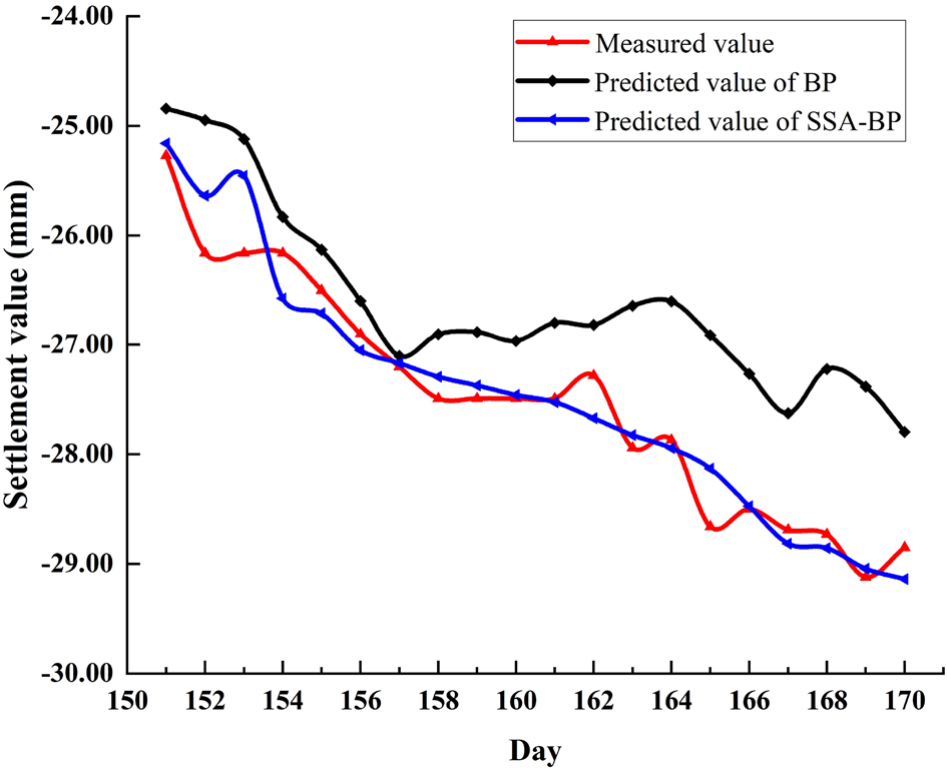
Comparison of BP, SSA-BP predicted value and measured value.

Figure 8 shows the comparison of predicted and measured values for BP, SSA-BP models. It can be seen from Fig. 8 that before day 157, the prediction accuracy of the optimized model was slightly higher than that of BP. After the 157th day, the predicted value of the BP model gradually deviates from the measured value, while the predicted value of SSA-BP closely matches the measured value.

In order to compare the prediction stability of the models, the variances of the predicted values of BP and SSA-BP models were calculated, and the results are shown in Table 2. BP model has a total of 20 predicted values, the first row of Table 2 represents the variance of the 1st to 10th predicted values, and the second row represents the variance of the 11th to 20th predicted values. The same is true for SSA-BP. By comparing the variance values, it can be seen that the stability of SSA-BP model is higher than that of BP model.

**Table 2:** Predicted value variance for BP and SSA-BP at P1.

BP	0.385	0.337	0.342	0.462	0.851	0.781	0.801	0.121	0.406	0.795
	0.136	0.205	0.816	0.568	0.652	0.704	0.797	0.835	0.951	0.943
SSA-BP	0.067	0.177	0.057	0.063	0.050	0.043	0.132	0.057	0.060	0.084
	0.114	0.049	0.085	0.074	0.101	0.064	0.109	0.161	0.090	0.136

For further comparison, the evaluation index values and average running time (The number of iterations in the paper is set to 1000) of the two models are listed in Table 3.

**Table 3:** Evaluation index value and average running time of BP, SSA-BP at P1.

Models	MAE (mm)	MAPE (%)	MSE (mm^2^)	RMSE (mm)	R^2^	Runtime (s)
BP	0.8786	3.1566	1.0092	1.0046	0.795	2
SSA-BP	0.2138	0.7874	0.0823	0.2868	0.937	96.3

From the data in Table 3, it can be seen that the five evaluation indicators of the optimized model have been improved to varying degrees after optimization, which proves that SSA has indeed exerted its optimization ability. The running time of SSA-BP has been greatly increased compared with BP, and the long solution time is indeed a major disadvantage of the optimization algorithm. But this runtime is acceptable relative to a dataset where the time unit is days. In summary, it can be seen that SSA can exert its excellent global search and local optimization capabilities to generate optimal weights and thresholds, thereby improving the prediction accuracy and generalization ability of the BP model.

**Figure 9:**
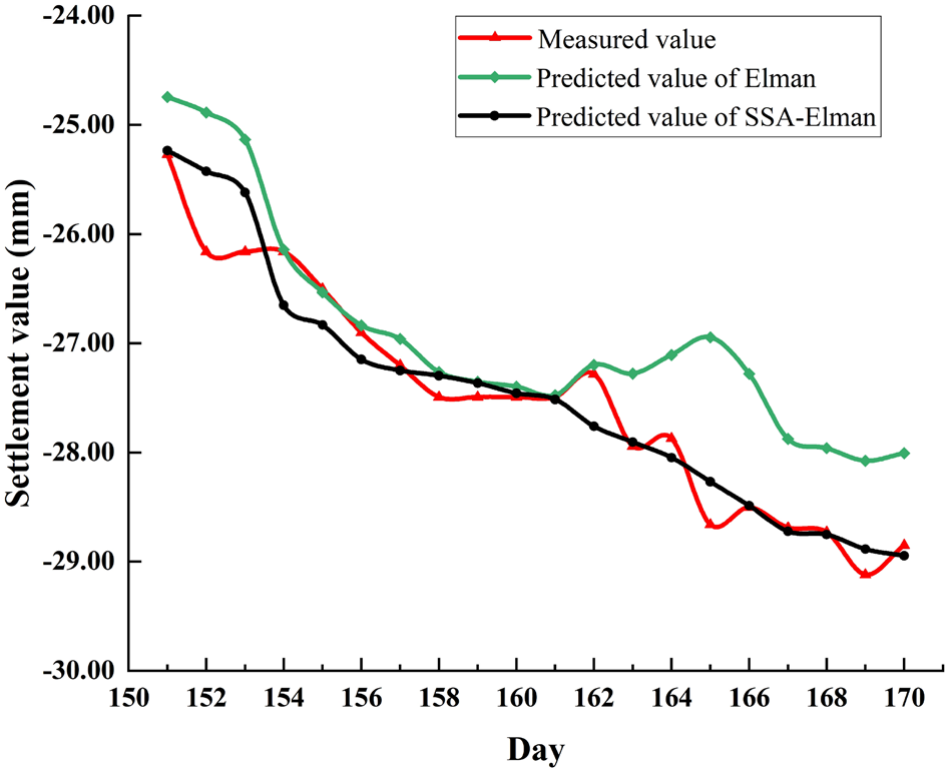
Comparison of Elman, SSA-Elman predicted and measured values.

Next, Elman and SSA-Elman models are selected to further demonstrate the optimization capability of SSA. The comparison between the predicted values and the measured value is shown in Fig. 9.

From Fig. 9, the prediction accuracy of the two models before the 161st day is similar, but after the 161st day, the predicted value curve of SSA-Elman fits the measured value curve better than that of Elman. Furthermore, the variance of the predicted values of the two models and related evaluation indicators are listed in Tables 4 and 5.

**Table 4:** Predicted value variance for Elman and SSA-Elman.

Elman	0.084	0.093	0.108	0.232	0.276	0.327	0.347	0.371	0.383	0.397
	0.408	0.429	0.459	0.469	0.486	0.585	0.699	0.711	0.770	0.803
SSA-Elman	0.067	0.177	0.057	0.063	0.050	0.043	0.132	0.057	0.060	0.084
	0.114	0.049	0.085	0.074	0.101	0.064	0.109	0.161	0.090	0.136

**Table 5:** Evaluation index value and average running time of Elman, SSA-Elman.

Models	MAE (mm)	MAPE (%)	MSE (mm^2^)	RMSE (mm)	R^2^	Runtime(s)
Elman	0.5794	2.084	0.5817	0.7627	0.774	3
SSA-Elman	0.2145	0.7938	0.0893	0.2988	0.925	190.8

From the comparison results in Table 4 that the variance of predicted value of SSA-Elman is lower than that of Elman, indicating that the stability of the model has been improved. From Table 5, it can be seen that the running time of the optimized model becomes longer, indicating that the solution time has increased significantly, while the changes of other evaluation indicators indicate that the optimized model has higher prediction accuracy and generalization ability.

### Verify the effectiveness of the joint model on the P2 monitoring point

**Figure 10:**
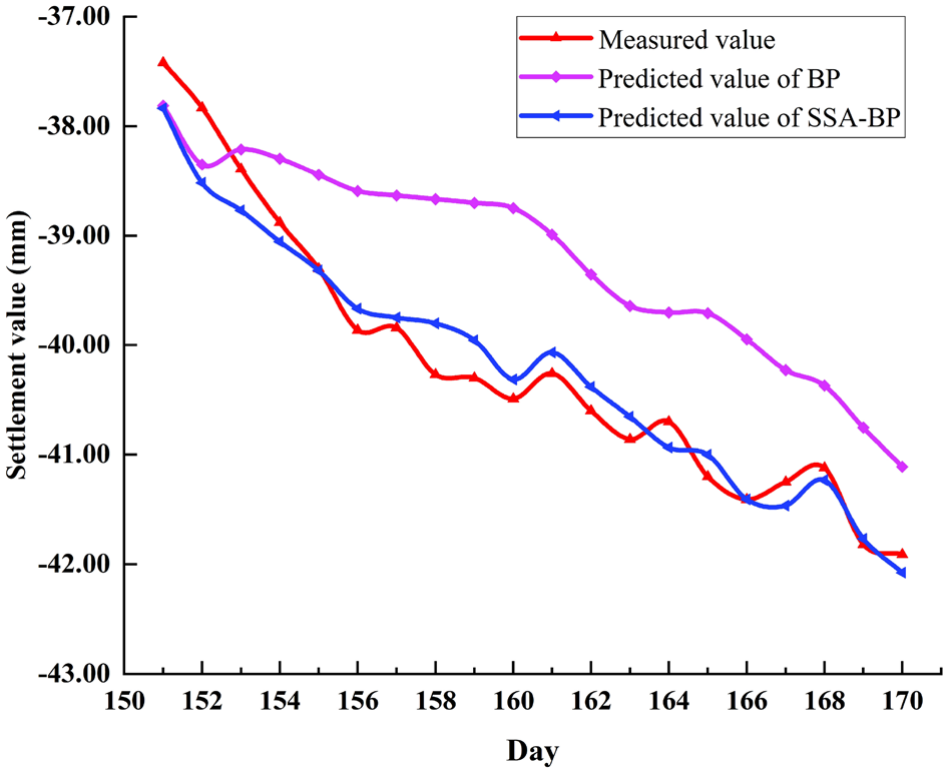
BP, SSA-BP predicted value and measured value at P2 point.

In order to verify the validity of joint model, the data of P2 monitoring points were selected for verification again. From Fig. 10, it can be seen that the predicted value curve of the optimized model is obviously better than the initial model in terms of prediction accuracy and curve fit. From the data comparison analysis in Tables 6 and 7, it can be concluded that the optimized model is better than the initial model in terms of stability and prediction performance.

**Table 6:** Predicted value variance of BP and SSA-BP at P2.

BP	0.019	0.139	0.141	0.187	0.224	0.326	0.347	0.347	0.367	0.268
	0.367	0.948	0.798	0.699	0.665	0.886	0.933	0.748	0.841	0.827
SSA-BP	0.051	0.021	0.069	0.024	0.072	0.016	0.017	0.014	0.022	0.348
	0.079	0.108	0.060	0.235	0.281	0.066	0.059	0.080	0.149	0.154

**Table 7:** Evaluation index value and average running time of BP, SSA-BP at P2.

Models	MAE (mm)	MAPE (%)	MSE (mm^2^)	RMSE (mm)	R^2^	Runtime(s)
BP	1.0639	2.6303	1.3079	1.1436	0.785	2
SSA-BP	0.2275	0.5743	0.0774	0.2783	0.954	90

**Figure 11:**
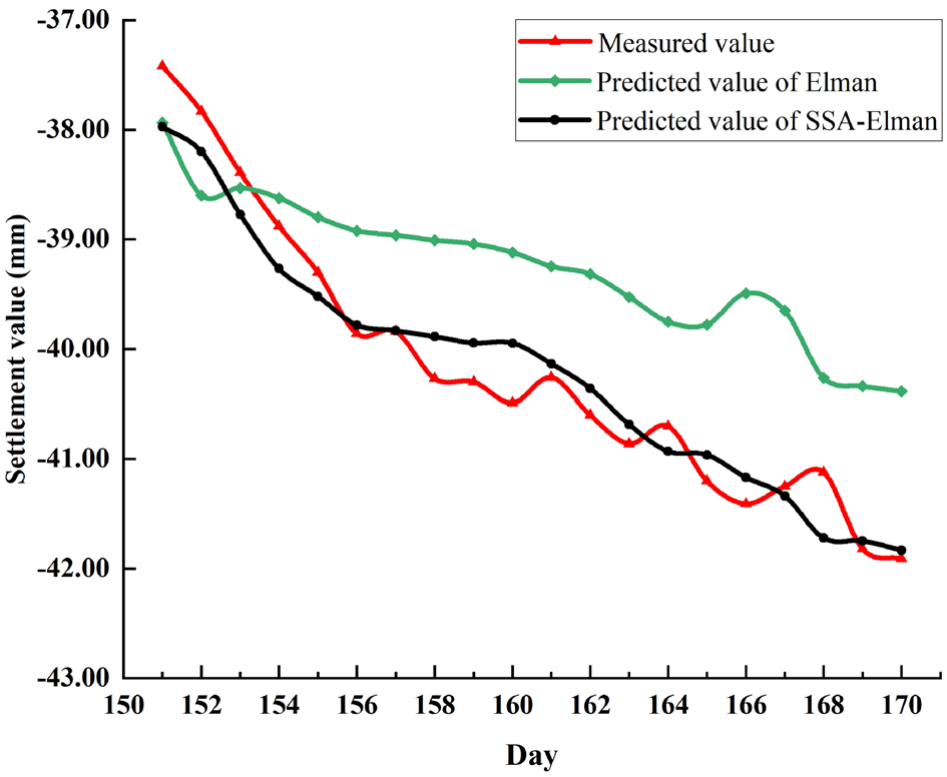
Elman, SSA-Elman predicted value and measured values at P2 point.

At point P2, the Elman model's predictive performance is similar to that of BP. As shown in Fig. 11, its predicted value curve deviates significantly from the measured value curve, which indicates that its generalization ability needs to be enhanced. While, the predicted value of the SSA-Elman model is relatively more fitting to the measured value.

The data in Tables 8 and 9 again demonstrate that the optimized model has better predictive performance.

**Table 8:** Predicted value variance of Elman and SSA-Elman at P2.

Elman	0.004	0.021	0.052	0.101	0.111	0.194	0.205	0.191	0.201	0.076
	0.065	0.150	0.182	0.112	0.104	0.011	0.298	0.194	0.287	0.139
SSA-Elman	0.014	0.019	0.010	0.029	0.030	0.025	0.034	0.033	0.035	0.028
	0.025	0.035	0.044	0.021	0.018	0.031	0.079	0.054	0.082	0.024

**Table 9:** Evaluation index value and average running time of Elman, SSA-Elman at P2.

Models	MAE (mm)	MAPE (%)	MSE (mm^2^)	RMSE (mm)	R^2^	Runtime(s)
Elman	1.0634	2.6199	1.3376	1.1566	0.843	4
SSA-Elman	0.2684	0.6741	0.1005	0.317	0.941	230.4

### Performance comparison between SSA and other optimization algorithms

**Figure 12:**
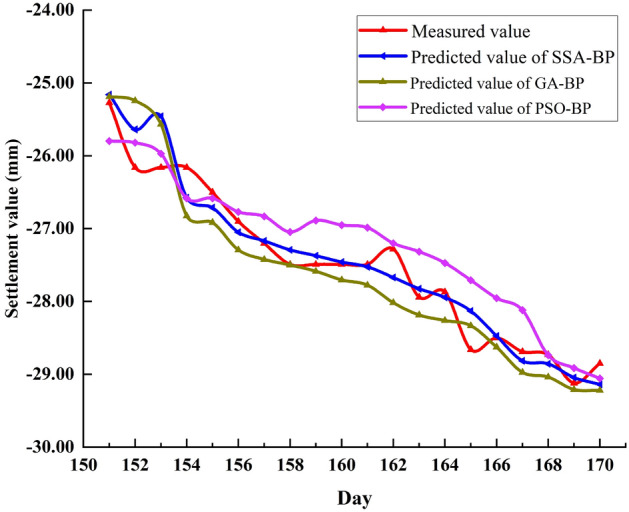
Comparison of predicted values of SSA-BP, GA-BP and PSO-BP at P1.

To further verify the superiority of SSA, other algorithms including Genetic Algorithm (GA) and Particle Swarm Optimization (PSO) were selected to optimize BP, and then the predicted values of P1 and P2 monitoring points were compared respectively. The predicted values of the three models at P1 are shown in Fig. 12. It can be seen that the predicted value curve of PSO-BP deviates significantly from the measured value curve after the 158th day. The predicted value curve of GA-BP deviates not much, but it is still not as good as that of SSA-BP.

To more directly compare the prediction performance of the three models, the variance of the predicted value of the models, the five evaluation indicators and the average running time are listed in Tables 10 and 11, respectively.

**Table 10:** Predicted value variance of SSA-BP, GA-BP and PSO-BP at P1.

SSA-BP	0.067	0.177	0.057	0.063	0.050	0.043	0.132	0.057	0.060	0.084
	0.114	0.049	0.085	0.074	0.101	0.064	0.109	0.161	0.090	0.136
GA-BP	0.055	0.042	0.058	0.198	0.239	0.335	0.378	0.463	0.117	0.139
	0.417	0.537	0.593	0.590	0.596	0.524	0.329	0.356	0.405	0.385
PSO-BP	0.286	0.295	0.304	0.353	0.387	0.406	0.415	0.237	0.458	0.458
	0.465	0.429	0.439	0.454	0.531	0.586	0.585	0.202	0.200	0.215

**Table 11:** Evaluation index value and average running time of three models at P1.

Models	MAE (mm)	MAPE (%)	MSE (mm^2^)	RMSE (mm)	R^2^	Runtime(s)
SSA-BP	0.2138	0.7874	0.0823	0.2868	0.937	96.3
GA-BP	0.3368	1.2456	0.1678	0.4097	0.902	45.5
PSO-BP	0.3871	1.4055	0.2021	0.4495	0.881	15

A comprehensive comparative analysis of the data in Tables 10 and 11 shows that the variance of predicted values of the three models is ranked from low to high as SSA-BP, GA-BP and PSO-BP. From the perspective of the five evaluation indicators of the model, SSA-BP is also the most Excellent, followed by GA-BP, and finally PSO-BP. In terms of average solution time, PSO-BP is much less time-consuming than the other models. But overall, the optimization efficiency of SSA is better than the other algorithms.

**Figure 13:**
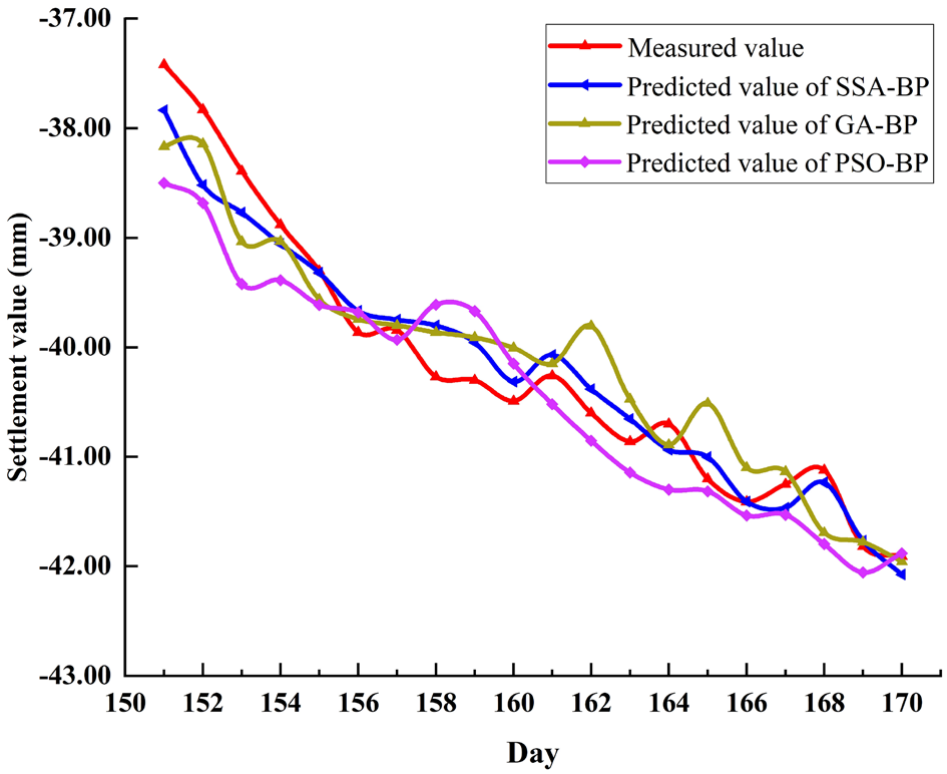
Comparison of predicted values of SSA-BP, GA-BP and PSO-BP at P2.

Select the data of the P2 monitoring point to verify again and the predicted results are plotted in Fig. 13. It can be seen that the predicted value curve of the GA-BP model started to fluctuate after day 162, whereas the predicted value curve of the PSO-BP model performed poorly before day 160. In conclusion, the fitness of the SSA-BP model is better than that of other models.

At point P2, the variance of the predicted values of the three models and the model evaluation index are shown in Tables 12 and 13 respectively.

**Table 12:** Predicted value variance of SSA-BP, GA-BP and PSO-BP at P2.

SSABP	0.051	0.021	0.070	0.024	0.072	0.016	0.017	0.014	0.022	0.348
	0.079	0.108	0.060	0.235	0.281	0.066	0.059	0.080	0.149	0.154
GA-BP	0.138	0.033	0.038	0.104	0.053	0.076	0.087	0.090	0.100	0.092
	0.162	0.067	0.330	0.191	0.106	0.203	0.247	0.420	0.419	0.302
PSO-BP	0.407	0.343	0.162	0.187	0.160	0.203	0.025	0.160	0.042	0.096
	0.092	0.161	0.198	0.179	0.177	0.231	0.046	0.060	0.122	0.123

**Table 13:** Evaluation index value and average running time of of three models at P2.

Models	MAE (mm)	MAPE (%)	MSE (mm^2^)	RMSE (mm)	R^2^	Runtime(s)
SSA-BP	0.2275	0.5743	0.0774	0.2783	0.954	90
GA-BP	0.3413	0.8553	0.1742	0.4174	0.894	47
PSO-BP	0.4266	1.0797	0.2744	0.5238	0.861	12

Comprehensively comparing and analyzing the data in Tables 12 and 13, at the P2 monitoring point, it can still be concluded that the optimization efficiency of SSA is better than other algorithms.

### Comparison of optimization performance of SSA for other kind of neural network

A Kernel Extreme Learning Machine (KELM) and Least Squares Support Vector Machine (LSSVM) were selected and their parameters were optimized using SSA Among them, the two parameters that KELM needs to optimize are the regularization coefficient and the kernel function parameter, and the two parameters that LSSVM need to optimize are the penalty factor and the kernel parameter. The data of P1 and P2 monitoring points are selected for prediction and comparison data are presented in Table 14.

**Table 14:** Evaluation indicators of four models under P1 and P2 monitoring points.

Models	MAE (mm)	MAPE (%)	MSE (mm^2^)	RMSE (mm)	R^2^	Runtime(s)
**P1 point**						
KELM	0.7856	2.827	0.7858	0.8865	0.856	3
SSA-KELM	0.313	1.1486	0.1762	0.4197	0.920	11
LSSVM	0.8874	3.2036	0.894	0.9455	0.914	3
SSA-LSSVM	0.3165	1.1602	0.1491	0.3861	0.93	12
**P2 point**						
KELM	1.1914	2.9153	1.9994	1.414	0.680	3
SSA-KELM	0.3399	0.8555	0.1843	0.4293	0.917	10
LSSVM	0.5841	1.485	0.5607	0.7488	0.730	2
SSA-LSSVM	0.3528	0.8913	0.1911	0.4372	0.882	15

Judging from the data of the P2 monitoring point in Table 14, it can be concluded that when the basic model itself does not perform well, the optimization effect of SSA is more significant; from the data of the P1 monitoring point, when the basic model itself has a good performance, SSA can still be able to achieve a small improvement of its prediction performance.

**Figure 14:**
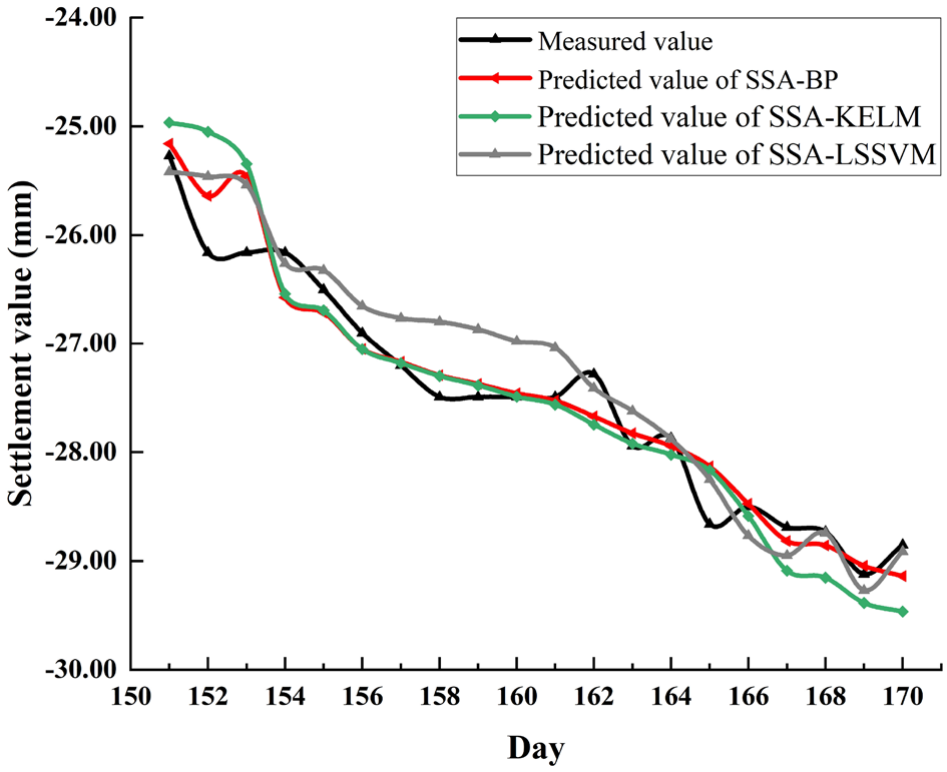
Comparison of predicted values of SAA-BP, SSA-KELM and SSA-LSSVM at point P1.

**Figure 15:**
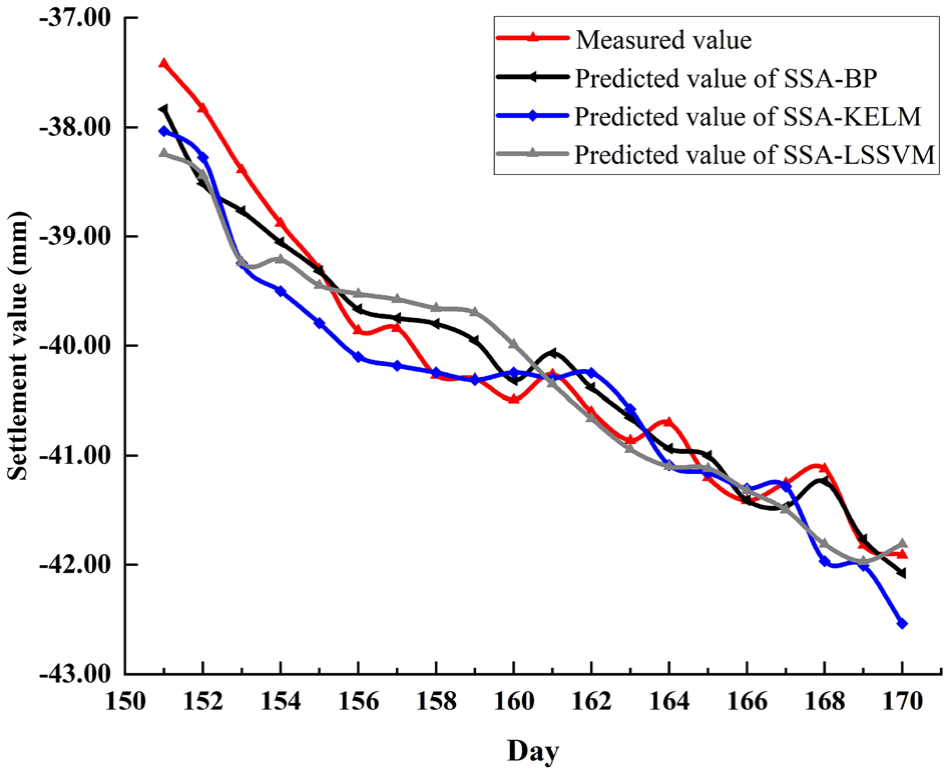
Comparison of predicted values of SAA-BP, SSA-KELM and SSA-LSSVM at point P2.

Figures 14 and 15 are the comparisons between the predicted and measured values of the three models at points P1 and P2, respectively. From Fig. 14, the SSA-LSSVM performs significantly worse than SSA-BP model from day 155 to day 161, while the SSA-KELM performs worse than SSA-BP model before day 153 and after day 167. Overall, the prediction performance of SSA-BP at the P1 monitoring point is slightly better than the other two optimization models. From Fig. 15, it is still the same conclusion at the P2 monitoring point.

Table 15 shows the evaluation index values of the three models at monitoring points P1 and P2 and their average solution time. At the two monitoring points P1 and P2, from the data in the table, although the solution time of SSA-BP is longer than the other models, the evaluation index of the SSA-BP model is slightly better than the other optimization models. Since the time unit of the dataset is day, the increase degree in runtime is perfectly acceptable by comparison. Therefore, comprehensive analysis shows that the performance of the joint model constructed by SSA and BP neural network is slightly better than the joint model constructed by SSA and other kind of neural network.

**Table 15:** Evaluation indicators of three models under P1 and P2 monitoring points.

Models	MAE (mm)	MAPE (%)	MSE (mm^2^)	RMSE (mm)	R^2^	Runtime(s)
**P1 point**						
SSA-BP	0.2138	0.7848	0.0823	0.2868	0.937	96.3
SSA-KELM	0.313	1.1486	0.1762	0.4197	0.920	11
SSA-LSSVM	0.3165	1.1602	0.1491	0.3861	0.93	12
**P2 point**						
SSA-BP	0.2275	0.5743	0.0774	0.2783	0.954	90
SSA-KELM	0.3399	0.8555	0.1843	0.4293	0.917	10
SSA-LSSVM	0.3528	0.8913	0.1911	0.4372	0.882	15

## Conclusion

In summary, the following conclusions can be drawn from this paper:

1. SSA has a significant effect on the optimization of gradient descent neural networks. After the BP and Elman neural networks selected in this paper are optimized by SSA, the prediction performance of the model is greatly improved, and its stability is also improved. Although the solution time of the optimization model is significantly increased, which is also a disadvantage of evolutionary algorithms, however, relative to the time unit of the dataset, this is acceptable.
2. This paper first selects the data of the P1 monitoring point for predictive analysis, and draws the conclusion in (1), and then selects the data of the P2 monitoring point again for prediction to verify its effectiveness. The result analysis shows that the optimization model established in this paper is effective and reliable.
3. In this paper, by conducting predictive analysis at point P1 and verifying its effectiveness at point P2, it can be concluded that SSA has more efficient optimization performance than GA and PSO algorithms. In general, the predictive performance of SSA-Gradient descent models outperforms other types of optimization models.
4. In this paper, in addition to the BP model, the Elman, KELM and LSSVM models are also selected for optimization with SSA, and then the prediction analysis and verification are carried out at the P1 and P2 monitoring points respectively. The data show that SSA can effectively optimize the above model and improve its prediction performance, and it also shows that the optimization efficiency of SSA for BP and Elman neural networks is slightly better than other kind of neural network.
5. The seven factors considered in this paper are feasible as input variables of the model. However, it is not certain that 7 is the optimal number of input parameters. Because, there are many factors that may affect the settlement around the foundation pit. Subsequent work will consider potential influencing factors in more detail and comprehensively, so as to make more reasonable and accurate predictions.

## Data Availability

The datasets generated during the current study are available from the corresponding author on reasonable request.
